# MP-VHPPI: Meta predictor for viral host protein-protein interaction prediction in multiple hosts and viruses

**DOI:** 10.3389/fmed.2022.1025887

**Published:** 2022-11-16

**Authors:** Muhammad Nabeel Asim, Ahtisham Fazeel, Muhammad Ali Ibrahim, Andreas Dengel, Sheraz Ahmed

**Affiliations:** ^1^Department of Computer Science, Technical University of Kaiserslautern, Kaiserslautern, Germany; ^2^German Research Center for Artificial Intelligence GmbH, Kaiserslautern, Germany

**Keywords:** virus-host protein-protein interaction, meta predictor, feature agglomeration, SARSCoV-2, Ebola virus, H1N1 virus

## Abstract

Viral-host protein-protein interaction (VHPPI) prediction is essential to decoding molecular mechanisms of viral pathogens and host immunity processes that eventually help to control the propagation of viral diseases and to design optimized therapeutics. Multiple AI-based predictors have been developed to predict diverse VHPPIs across a wide range of viruses and hosts, however, these predictors produce better performance only for specific types of hosts and viruses. The prime objective of this research is to develop a robust meta predictor (MP-VHPPI) capable of more accurately predicting VHPPI across multiple hosts and viruses. The proposed meta predictor makes use of two well-known encoding methods Amphiphilic Pseudo-Amino Acid Composition (APAAC) and Quasi-sequence (QS) Order that capture amino acids sequence order and distributional information to most effectively generate the numerical representation of complete viral-host raw protein sequences. Feature agglomeration method is utilized to transform the original feature space into a more informative feature space. Random forest (RF) and Extra tree (ET) classifiers are trained on optimized feature space of both APAAC and QS order separate encoders and by combining both encodings. Further predictions of both classifiers are utilized to feed the Support Vector Machine (SVM) classifier that makes final predictions. The proposed meta predictor is evaluated over 7 different benchmark datasets, where it outperforms existing VHPPI predictors with an average performance of 3.07, 6.07, 2.95, and 2.85% in terms of accuracy, Mathews correlation coefficient, precision, and sensitivity, respectively. To facilitate the scientific community, the MP-VHPPI web server is available at https://sds_genetic_analysis.opendfki.de/MP-VHPPI/.

## 1. Introduction

Viruses have a long history of posing threat to living organisms (1) as they have caused more than 300 million deaths worldwide (2). A recent emanation Coronavirus) is an example of an acute virus that caused a global pandemic (3). According to World Health Organization, Coronavirus has caused approximately more than 400 million infections and 6 million deaths across the globe (4). Similarly, the Ebola virus was also responsible for an epidemic that caused more than 11 thousand deaths in Africa (5).

Viruses are small microscopic particles that contain genetic material (DNA or RNA) surrounded by a protein coat (1). These particles are considered non-living because of their inability to reproduce or perform any other biological function since they lack specific proteins (6). However, once they get a chance to enter inside the host cell, they make interactions with available proteins in the cell and become capable to reproduce themselves (7). Initially, to enter inside a host cell, the viruses interact with the host cell receptor proteins (8) and replicate themselves by injecting their genetic material into the cell's genome (9). After the entrance into the cell, the aim of viruses is to interact with diverse types of proteins through which they can control the process of the cell cycle, particle assembly, apoptosis, and cell metabolism (7, 10). The relationships between host and virus proteins are termed virus-host protein-protein interactions (11).

To prevent viruses from interacting with host proteins, hosts have sophisticated mechanisms to recognize and confine the viruses, such as the dendritic and β- cells, T-cells, and major histocompatibility complex (12). Therefore, viruses tend to adapt in an efficient manner by interacting with specific host proteins and cellular pathways that prove to be substantial for evading or inactivating factors that are detrimental to viral growth (7). Meanwhile, to enhance immunity against viruses, it is difficult to develop efficient vaccines/drugs because of the poor understanding of different mechanisms that have been adapted by the viruses, and their frequent transmissibility from cell-to-cell or species-to-species (13). Consequently, analyzes of virus-host PPIs are essential to explore their effects on diverse types of biological functions and to design antiviral strategies (14). Furthermore, through such analyzes essential viral proteins and viral dependencies on host proteins can be identified as drug targets to halt the replication process of viruses by pharmacological inhibition (15).

Multiple experimental techniques have been utilized to identify virus-host protein-protein interactions (VHPPIs) such as protease assay (16), surface plasmon resonance (SPR) (17), Förster resonance energy (FRET) (18), Yeast two hybrid screening (Y2H) (19) and affinity purification mass spectrometry (AP-MS) (20). Such conventional wet lab methods are expensive, time-consuming, and error-prone, which impede inter and intra species large scale proteomics sequence analyzes. To overcome the shortcomings of experimental approaches, the development of machine learning applications for efficient proteomics sequence analyzes across different species (e.g., humans, viruses) is an active area of research (21–23). Researchers have developed a machine learning based clustering applications to distinguish several microbial pathogens (24, 25) and classification applications to categorize the genes associated with the survival of pathogens under certain environmental conditions, antibiotics, or other disturbances (26). Similarly, researchers have developed classification applications to determine VHPPIs that play a key role in understanding the functional paradigms of viruses as well as host responses (27, 28). With an aim to provide cheap, fast, and accurate virus-host protein-protein analyzes, to date, around 13 AI-based predictors (21–23, 27–36) have been proposed.

Recently, Yang et al. (36) proposed a VHPPI predictor by utilizing position-specific scoring matrices to statistically represent virus and host protein sequences that were further passed to Siamese convolutional neural network (CNN) for VHPPI prediction. The predictor was evaluated on VHPPI data of human proteins and 8 different viruses. Another similar predictor namely, Deep Viral (35) used one hot vector encoding (OHE) for the discretization of sequences and convolutional neural network architecture for VHPPI prediction. Deep Viral was evaluated on VHPPIs of humans and 12 different viruses. Deep-VHPPI (27) predictor also used OHE and attention mechanism along with CNN for VHPPI prediction. The predictor was evaluated on VHPPI data related to humans and 4 different viruses.

Ding et al. (34) proposed a VHPPI predictor based on long short-term memory (LSTM) neural network. At preprocessing stage, they generated statistical representations of viral and host proteins by reaping the benefits of 3 different encoders namely, the relative frequency of amino acid triplets (RFAT), frequency difference of amino acid triplets (FDAT), and amino acid composition (AC). The predictor (34) was evaluated on VHPPIs across proteins belonging to 137 different viruses and 13 hosts. Denovo (29) used amino acid properties such as dipoles and volumes of side chains to represent 20 amino acids (AAs) with only 7 cluster numbers to reduce the diversity of amino acids. The sequences were then encoded based on the normalized kmer frequencies of 7 unique clusters. Denovo predictor used SVM classifier and was evaluated on the dataset of 10 viruses and human proteins. HOPITOR (37) used a similar encoding method as Denovo (29). HOPITOR used an SVM classifier and was evaluated on 10 different viruses and human proteins. Yang et al. (31) proposed InterSPPI-HVPPI which utilized Doc2vec embeddings and random forest (RF) classifier for VHPPI prediction. The predictor (31) was evaluated on data related to 12 viruses, and human proteins. Karabulut et al. (28) proposed meta predictor (ML-AdVInfect) that reaped the benefits of 4 existing predictors namely HOPITOR (37), InterSPPI-HVPPI (31), VHPPI, and Denovo (29). Specifically, the authors passed the predictions of existing predictors to the SVM classifier for the final VHPPI prediction.

Barman et al. (22) proposed a VHPPI predictor that utilized an RF classifier and statistical vectors generated through 4 different encoding methods namely, average domain-domain association score, virus methionine, virus seline, and virus valine. The predictor was evaluated on VHPPI data related to human proteins and 5 different viruses. Zhou et al. (30) used 7 sequence encoding methods i.e., RFAT, FDAT, AC, composition, transition, and distribution of amino acid groups. The approach (30) used an SVM classifier for VHPPI predictions across the proteins of 332 viruses and 29 hosts. Alguwaizani et al. (32) combined statistical vectors of 4 different encoders namely, amino acid repeats, the sum of the squared length of single amino acid repeats (SARs), maximum of the sum of the squared length of SARs in a window of 6 residues, and composition of amino acids in 5 partitions of the protein sequence. The predictor used an SVM classifier and experimentation was performed on VHPPI data related to 6 hosts and 5 viruses. Recently, Asim et al. proposed an LCGA-VHPPI predictor (38), that made use of a local-global residue context aware sequence encoding scheme and a deep forest model. The authors evaluated their predictor on data related to 23 viruses and human proteins.

Following the success of neural word embedding approaches in natural language processing and bioinformatics, Tsukiyama et al. proposed LSTM-PHV (21) that transformed viral host protein sequences to statistical vectors by learning statistical representation of k-mers in an unsupervised manner using Word2vec approach. The study (21) used bidirectional LSTM for VHPPI prediction and data of proteins belonging to 332 viruses and 29 hosts. Similarly, MTT (23) predictor utilized randomly initialized embeddings and LSTM based classifier. MTT predictor was evaluated on data related to 16 viruses and human proteins. Hangyu et al. (33) developed a VHPPI predictor based on Node2vec and Word2vec embeddings methods and a multilayer perceptron (MLP) classifier. Authors performed experimentation over 7 variants of the SARS virus and 16 different host proteins.

The working paradigm of existing VHPPI predictors can be broadly categorized into two different stages. In the first stage, raw sequences are transformed into statistical vectors where the aim is to capture distributional information of 21 unique amino acids. In the second stage, a machine or deep learning classifier is utilized to discriminate interactive viral-host protein pairs from non-interactive ones.

In the first stage, while transforming raw sequences to statistical vectors, 2 predictors (27, 35), make use of one hot vector encoding method which lacks information related to correlations of amino acids. Moreover, 3 predictors use word embedding generation approaches (21, 23, 35), that capture kmer-kmer associations but lack information related to the distribution of amino acids. To capture distribution and various patterns of amino acids, other predictors utilized 10 different mathematical encoders (22, 29, 31, 34, 36) however, these methods do not capture sequence order or amino acids correlation information. Such information is crucial for the analyzes of protein sequences as reported in the existing studies (39–42) which include sequence encoders such as APAAC and QS order. Despite the promising performance shown by APAAC and QS order encoders for subcellular location prediction (39), Cyclin protein classification (40), and protein-protein interaction prediction (41, 42) tasks, no researcher has explored their potential to effectively generate numerical representations of viral-host protein sequences.

In the second stage, 4 predictors (27, 34–36) utilize CNNs, 2 predictors (21, 34) make use of LSTM architecture and 8 predictors (22, 23, 28–33) use traditional classifiers. As such predictors have shown better performances across limited hosts and viruses, therefore these predictors cannot be generalized across multiple hosts and viruses. For instance, LSTM-PHV is the most recent predictor which managed to produce better performance for human and Coronavirus related VHPPI, but failed to produce similar performance over Zhou et al. (43) datasets that contain multiple hosts and viruses. To make a generic predictor capable of accurately predicting interactions across multiple hosts and viruses, only one meta predictor (28) has been developed. However, this meta-predictor relies on the predictions of 4 existing VHPPI predictors that have their own drawbacks at the sequence encoding and classification level.

With an aim to develop a more accurate and generic meta predictor, the contributions of this paper are manifold, i) It makes use of two different physicochemical properties-based sequence encoding methods namely, APAAC and QS order. In addition, unlike other protein sequence analysis tasks where numerical representations of complete raw protein sequences have been generated through these encoders by utilizing a combination of different physicochemical properties, the paper in hand proposes an effective way to generate numerical representations by using a precise subset of physicochemical properties. ii) Considering different physicochemical properties in both encoders extract some irrelevant and redundant features, to remove such features, it transforms the original feature space into a reduced and more discriminative feature space by utilizing a dimensionality reduction method named feature agglomeration. iii) Using separate and combined statistical vectors generated through APAAC and Qsorder, it generates more effective and discriminative probabilistic feature space by fusing the predictions of two different classifiers. Optimized probabilistic feature space is used to feed the SVM classifier which makes final predictions. iv) Large-scale experimentation over 7 public benchmark datasets and performance comparison of the proposed meta predictor with existing predictors is performed. v) To facilitate researchers and practitioners, a web application based on the proposed meta predictor is developed.

## 2. Materials and methods

This section briefly describes the working paradigm of the proposed predictor, benchmark datasets, and diverse types of evaluation measures.

### 2.1. Proposed meta-predictor

Machine learning classifiers cannot directly operate on raw sequences due to their dependency on statistical representations. While transforming raw protein sequences into statistical vectors, the aim is to encode positional and discriminative information about amino acids. To represent viral and host protein sequences by extracting both types of information, the proposed meta predictor makes use of two sequence encoders namely APAAC and QS order. The statistical vectors generated by these methods depend on certain physicochemical properties. For example, the APAAC (44) encoder contains three different physicochemical properties namely hydrophobicity, hydrophilicity, and side chain mass whereas, QS order (39) has two content matrices namely, Schneider and Grantham. However, it is important to investigate which particular properties of both encoders are appropriate in order to generate more comprehensive statistical vectors, rather than utilizing all the available properties.

To fully utilize the potential of both encoders, a strategy similar to the forward feature selection method is adopted to find out the most appropriate physicochemical properties. For instance, from 3 the properties of the APAAC encoder, first, we generate statistical vectors by using one property and compute the performance of the RF classifier. Similarly, we repeat the same process for the second and third properties in order to record the performance of the RF classifier. On the basis of higher performance, we take the property-specific statistical vectors and combine them with the second best performing property vectors. This is followed by the evaluation on the basis of combined features, if this does not yield any performance gains then the iterative process stops, and individual property-based statistical vectors with the highest performance are selected. In contrast, if there are any performance gains with such combinations then the combined encodings are retained and utilized further. A similar procedure is used to generate statistical representations using QS order.

The statistical vectors generated from the encoders may contain irrelevant and redundant features. In order to remove such features and retain only the most informative features, we utilize a dimensionality reduction algorithm named feature agglomeration (45). While reducing the dimensions of the original feature space, it is important to find the target dimension of the reduced feature space. To find an appropriate feature space, we reduce the dimension of the original feature space from 40 to 95% with a step size of 5%. By utilizing RF classifier based on its performance, we chose the most appropriate feature space. It is noteworthy to mention that the process of property selection and appropriate reduced feature space selection is performed only using training data.

In the current study, the training of meta-predictor can be seen as a two-stage process. In the first stage, the statistical vectors generated for virus-host protein sequences using APAAC and QS order are separately passed through two machine learning classifiers i.e., RF and ET (46). Then the prior representations are concatenated and passed again through the RF and ET classifiers, predictions of both classifiers using individual and combined encodings are utilized to create a new feature space on which the SVM classifier is trained to make final predictions.

**Figure 1:**
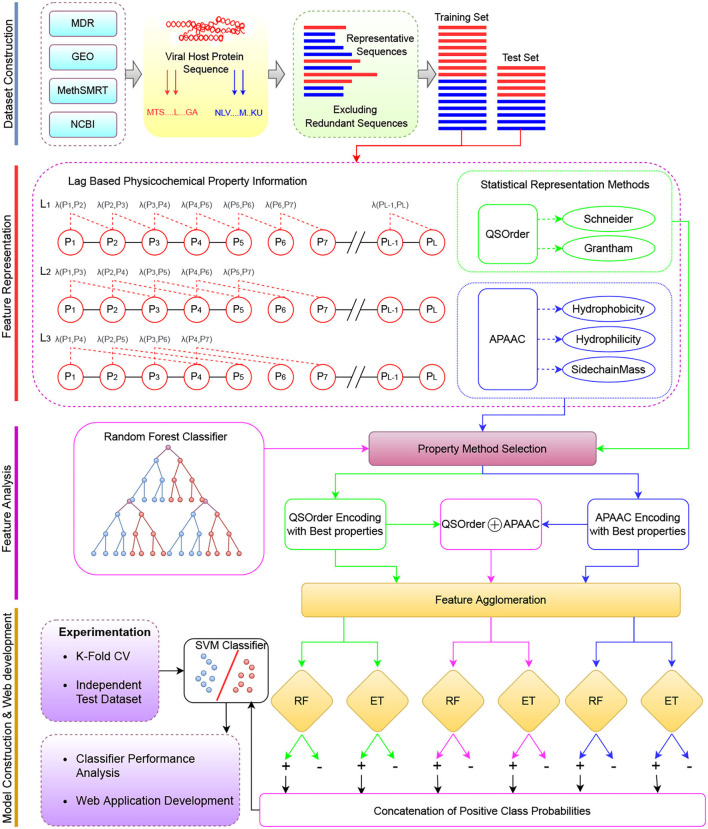
The overall working paradigm of the proposed VHPPIs predictor. **Dataset Construction** To begin with, different datasets are collected from existing studies based on VHPPIs from several databases such as HPID, Intact, and VirusMentha. **Feature Representation** Obtained protein sequences are encoded on the basis of two physicochemical properties based protein sequence encoders i.e., QS order and APAAC. **Feature Analyzes** Appropriate physicochemical properties are selected for the APAAC and QS order on the basis of feature analyzes. **Model Construction** the VHPPIs predictor is an SVM model formed on the basis of probabilistic vectors obtained from the RF and ET classifiers. Finally, a web server is established for fast, and easy on-go analyzes of VHPPIs.

Figure 1 describes a graphical illustration of the proposed meta predictor's workflow. The more detailed working of the encoding methods is given in Section 2.1.1. The dimensionality reduction method is explained in Section 2.1.2. In addition, details of the machine learning classifiers are provided in Section 2.3.

#### 2.1.1. Protein sequence encoding

The following subsections briefly illustrate the working paradigm of APAAC and QS order sequence encoding methods.

##### 2.1.1.1. Amphiphilic Pseudo-Amino Acid Composition (APAAC)

Chou (44, 47) proposed an APAAC encoder that makes use of pre-computed physicochemical values of hydrophobicity, hydrophilicity, and side chain mass (44, 47). Each physicochemical property contains 20 float values associated with 20 unique amino acids (Supplementary Table S1). These values are computed based on diverse types of information related to protein folding, and protein's interactions with the environment and other molecules. For each of the three quantitative properties, the values of its corresponding amino acids are normalized to zero mean and unit standard deviation through Equation 1.


(1) $$f(x)=\{\begin{array}{l} Mean[p_{i}]=\frac{\sum_{k=1}^{20}p_{i}[AA_{k}]}{20},\\ S[p_{i}]=\sqrt{\frac{(\sum_{k=1}^{20}{\left(p_{i}[AA_{k}]-Mean[p_{i}]\right)}^{2})}{20}},\\ P[p_{i}]=\frac{p_{i}[AA_{k}]-Mean[p_{i}]}{S[p_{i}]},\;\\ k\in \{1.,2,3,\cdots ,20\},\\ p_{i}\in \{\text{hydrophobicity, hydrophilicity, side chain mass}\}. \end{array}$$

Whereas, *p*_*i*_ represents the physicochemical property based value of an amino acid (*AA*_*k*_) which is either hydrophobicity, hydrophilicity, or side chain mass. In Equation 1, Mean[*p*_*i*_] is the mean of 20 amino acids in each property, and S[*p*_*i*_] is the standard deviation, where both can be computed using Equation 1.

In each physicochemical property, using normalized values of all 20 amino acids, the order of amino acids within the host and viral protein sequences is captured using a lag-based phenomenon.

For instance, we have a raw sequence S=*R*_1_, *R*_2_, *R*_3_, *R*_4_, ⋯ , *R*_*L*_, where *R*_1, ⋯ , *L*_ denotes 20 unique amino acids. If lag=1, then two most contiguous amino acids i.e., *S*_*lag*1_ = *R*_1_*R*_2_, *R*_2_*R*_3_, *R*_3_*R*_4_, *R*_4_*R*_5_, are taken, for lag=2, second-most contiguous amino acids, i.e., *S*_*lag*2_ = *R*_1_*R*_3_, *R*_2_*R*_4_, *R*_3_*R*_5_ are taken by skipping 1 amino acid, and for lag=3, third-most contiguous amino acids are taken by skipping 2 amino acids i.e., *S*_*lag*3_ = *R*_1_*R*_4_, *R*_2_*R*_5_, and so on. After generating bigrams, from *S*_*lag*1_, *S*_*lag*2_, and *S*_*lag*3_, iteratively, bigrams are taken and in each bigram, physicochemical values of both amino acids are multiplied using a correlation function shown in Equation 2.


(2) $$\begin{array}{l} \begin{array}{c} P_{i}\left[B\right]=p_{i}\left(AA_{j}\right).p_{i}\left(AA_{k}\right),\\ p_{i}\in \left\{\text{hydrophobicity, hydrophilicity, side chain mass}\right\} \end{array} \end{array}$$

After computing the correlation functions, for a property, across N number of lags, a single float is computed by averaging property values across all the lag-based amino acid bigrams.


(3) $$\begin{array}{l} Enc\left[p_{i}\right]=\overset{lag}{\underset{l=1}{\sum }}\frac{P\left[B\right]}{seqlen-lag_{l}}. \end{array}$$

Furthermore, both types of sequence order and amino acid distributional information can be captured using Equation (4).


(4) $$\begin{array}{l} \begin{array}{c} Enc\left[AA\right]=\frac{frequencyofAAinproteinsequence}{1+w\times Enc\left[p_{i}\right]}, \end{array} \end{array}$$

Here, *w* is a weight parameter that varies from 0.1 to 1. Similarly, normalization is applied to the original sequence order information by using Equation (5),


(5) $$\begin{array}{l} Enc\left[p_{i}\right]lag_{i}=\frac{w\times Enc\left[p_{i}\right]lag_{i}}{1+w\times Enc\left[p_{i}\right]}. \end{array}$$

Once the amino acid distribution and sequence order related information are encoded, the final statistical representation is obtained by concatenating the amino acid distributions and correlations among amino acids, that represent the sequence order information of a protein sequence.


(6) $$\begin{array}{l} Encoding\left[seq\right]P_{i}=Enc\left[AA\right]∥Enc{\left[p_{i}\right]}_{lag_{i}} \end{array}$$

The dimension of the final statistical vector for a single physicochemical property is 20 + lag-D vector and for 3 physicochemical properties, the final statistical vector is (20 + lag) × 3 dimensional vector. As a result, the first 20 numbers are the normalized amino acid frequencies and the next following discrete numbers reminisce the amphiphilic amino acid correlations along a protein chain.

##### 2.1.1.2. Quasi-sequence (QS) order

Owing to similar ideas like APAAC, QS order also encodes the sequence order and discriminative information based on different physicochemical properties (39). To incorporate a more significant sequence order information, QS order makes use of pre-computed values of 4 different physicochemical properties namely, hydrophobicity, hydrophilicity, polarity, and side chain volume to compute the coupling factors among the amino acids of a protein sequence (39). These physicochemical properties describe protein folding and its structural features, particularly surface physical chemistry. These pre-computed values have been averaged and on the basis of Manhattan distance, new values (20 × 20 = 400) have been provided by Schneider et al. (48) and Grantham et al. (49) (for details refer to Supplementary Tables S2, S3).

In QS order, first, the bigrams of amino acids are generated on the basis of a lag value that is quite similar to stride size in CNN. To compute a coupling factor *P*[*B*], distance values between two amino acids are taken from Supplementary Tables 2, 3, with respect to bigrams generated *via* lag value. The coupling factor *P*[*B*] can be written as;


(7) $$\begin{array}{l} \begin{array}{c} P\left[B\right]=D_{i}^{2}\left(AA_{k},AA_{j}\right),\\ D_{i}\in \left\{Schneider,Grantham\right\}, \end{array} \end{array}$$

where D is the distance value taken from the Schneider or Grantham's content matrices and *B* denotes a bigram of amino acids. Corresponding encoding value for a lag can be computed by averaging all the physicochemical distance values for bigrams,


(8) $$Encoding\;{\left[D_{i}\right]}_{lag_{i}}=\frac{\sum_{k=1}^{len\;seq-i}\left(P{\left[B\right]}_{k}\right)}{len\;seq-1}.$$

To get a single float value for the encoding, lag values are averaged depending on the size of the lag. For example, for lag=3, first, the bigrams are generated with lag=1,2,3, then the corresponding encodings for these bigrams are generated and averaged using the following equation.


(9) $$\begin{array}{l} Encoding\left[D_{i}\right]=\overset{lag}{\underset{i=1}{\sum }}Encoding{\left[D_{i}\right]}_{lag_{i}} \end{array}$$

These computed encoding values are normalized along with a weight factor *w*,


(10) $$\begin{array}{l} Encoding{\left[D_{i}\right]}_{lag_{i}}=\frac{w\times Encoding{\left[D_{i}\right]}_{lag_{i}}}{1+w\times Encoding\left[D_{i}\right]} \end{array}$$

To incorporate the distribution of amino acids, normalized frequencies of 20 different amino acids are computed, according to the following equation,


(11) $$\begin{array}{l} Encoding\left[AA_{k}\right]=\frac{frequencyofAA_{k}inproteinsequence}{1+w\times Encoding\left[D_{i}\right]}. \end{array}$$

Finally, (20+lag) × 2 dimensional statistical vector is formed by concatenating 20 amino acids distribution values and lag number of correlation factors referring to sequence order information with respect to distance values provided by Schneider and Grantham.


(12) $$\begin{array}{l} Encoding\left[seq\right]=Encoding\left[AA\right]∥Encoding{\left[D_{i}\right]}_{lag_{i}}, \end{array}$$

where, *Encdoing*[*AA*] represents the normalized frequency values of 20 different amino acids, and *Encoding*[*D*_*i*_]*lag*_*i*_ refers to the sequence order information.

#### 2.1.2. Dimensionality reduction *via* feature agglomeration clustering

Hierarchical clustering (HC) is a known group of clustering algorithms that construct clusters on the basis of similarities among the data samples. The end goal of HC is to compute clusters that are completely different from each other and data samples within a single cluster are similar to each other. Similar ideas are inherited by feature agglomeration, where the grouping is applied to the features of the data rather than the data samples. In feature agglomeration, two steps are iteratively followed to achieve the required dimensions of feature space namely, distance computation and pooling. First the distance among all the features is computed using Euclidean or Manhattan distance (50). On the basis of the minimum distance, two features are combined together on the basis of a pooling function which can be the mean of respective features. This process is repeated unless the features are reduced to desired dimensions.

#### 2.1.3. Iterative representation learning

Iterative representation learning is a crucial step for performance improvements of ML models, inspired by layer-wise training of deep learning models. In the current study, the proposed meta-predictor works in a two-stage process based on iterative representation learning. In the first stage, the statistical vectors generated for virus-host protein sequences by APAAC and QS order are separately passed through two machine learning models i.e., RF and ET. Then the prior representations are concatenated and passed again through the RF and ET classifiers. As a result, for protein sequences, in total around 6 different positive class probabilities are obtained. In the second stage, these probabilistic values are concatenated with each other to form a new 6-D feature vector for protein sequences. This probabilistic feature representation of protein sequences is used as an input for a support vector machine classifier that provides results for the prediction of VHPPIs.

### 2.2. Benchmark datasets

In order to develop and evaluate AI-based predictors for virus-host protein-protein interaction prediction, several datasets have been developed in the existing studies (11, 21, 22, 30, 32, 36). We have collected 7 publicly available benchmark datasets from 4 different studies. These datasets have been extensively utilized in the development/evaluation of the most recent VHPPI predictors (22, 29, 30, 36).

One dataset is taken from the study of Barman et al. (22), which contains VHPPIs across human and 4 viruses i.e., HIV-1, simian virus 40 (SV40), HBV, HCV, papilloma virus, these VHPPIs were downloaded from VirusMint database (51). Whereas, negative samples were collected from Uniprot (52) based on their dissimilarity with the true VHPPIs.

Similarly, another dataset is taken from Fatma et al. (29) work, which contains VHPPIs of humans and 173 viruses i.e., Paramyxoviridae, Filoviridae, Bunyaviridae, Flaviviridae, Adenoviridae, Orthomyxoviridae, Chordopoxviridae, Papillomaviridae, Herpesviridae, Retroviridae. These VHPPIs were collected from VirusMetha (53), and Uniprot (52). Negative class samples were generated by a random dissimilarity algorithm, which assumed the condition that two viral proteins comprised of similar amino acid sequences could not interact with the same host protein. The similarity between two proteins was decided through distance (dissimilarity) score based on normalized global alignment bit scores. Furthermore, once unique viral proteins were obtained, their interactions were decided based on the dissimilarity (distance) score >0.8 with host proteins.

The coronavirus and human proteins related dataset are taken from Yang et al. (36) work, where the interactions were collected from HPID (54), VirusHostNet (55), PHISTO (56), and PDB (57) databases. Moreover, negative samples were generated by dissimilarity-based negative sampling across the PPIs retrieved from Uniprot (36, 52).

To make the predictor generic and capable to predict interactions over new viruses, we collected 4 datasets from Zhou et al. (30) study. These datasets contain interactions related to 29 different hosts and 332 different viruses. To collect raw sequences and interactions, authors utilized 5 different databases namely PSICQUIC (58), APID (59), IntAct (60), Mentha (53), and Uniprot (52). Furthermore, for negative data, authors obtained protein sequences of 4 major hosts namely, human, non-human animal, plant, and bacteria, from UniProt (52), and removed sequences with a sequence similarity higher than 80% to any positive data using CD-HIT-2D (61). Moreover, in order to assess the applicability on new/unseen viruses, authors distributed VHPPIs of 29 hosts and 332 viruses into 4 different train and 2 test sets, the distribution of viruses and hosts in these datasets is given below,

**TR1:** PPIs between human and any virus except H1N1 virus.
**TR2:** PPIs between human and any virus except Ebola virus.
**TR3:** PPIs between any host and any virus except H1N1 virus.
**TR4:** PPIs between any host and any virus except Ebola virus.
**TS1:** PPIs between human and H1N1 virus.
**TS2:** PPIs between human and Ebola virus.

**Figure 2:**
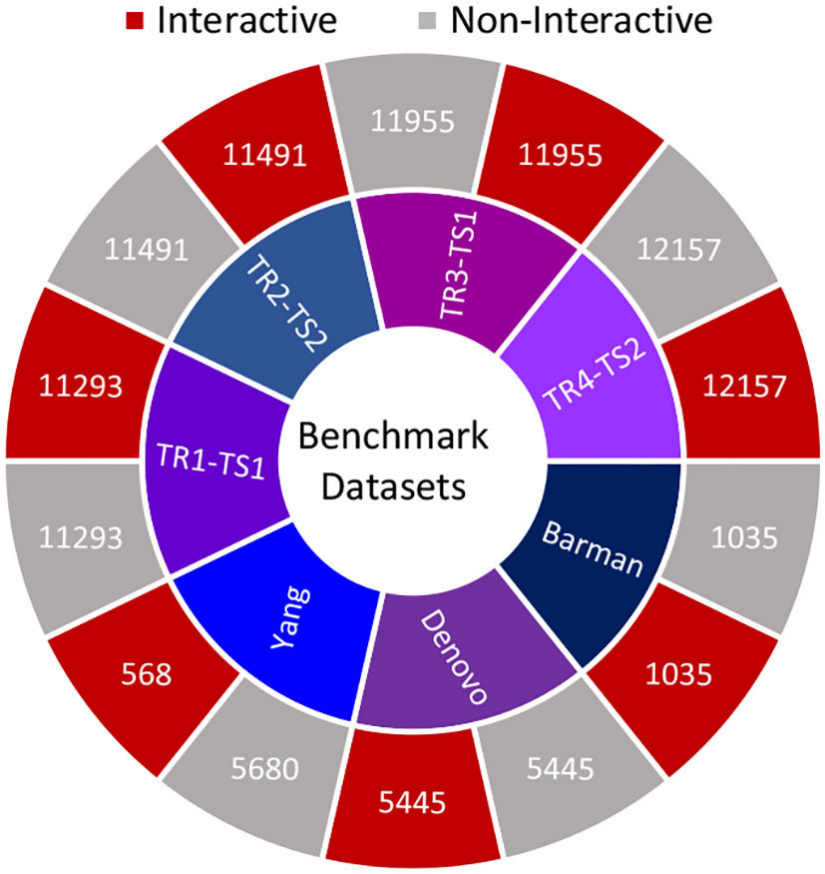
Summary statistics of 7 datasets utilized in this study. For each study, the respective number of positive and negative samples are shown.

Furthermore, Figure 2 summarizes the statistics of datasets in terms of the number of positive and negative samples. In order to perform experimentation, selected datasets are more appropriate due to multiple reasons such as recent VHPPI predictors reporting their performance scores, making it possible to compare our proposed VHPPI predictor to existing predictors directly. These datasets contain sufficient VHPPIs which enable training machine learning models in an optimal way. Furthermore, these datasets contain diverse VHPPIs across a broad selection of viruses and hosts which allows testing the generalizability of the model against multiple hosts and viruses for the task of VHPPI prediction.

### 2.3. Virus-host protein-protein interaction prediction

The following section summarizes the machine learning (ML) classifiers used to predict virus-host protein-protein interactions.

#### 2.3.1. Support vector machine

SVM classifier finds hyperplane(s) in an N-dimensional feature space that can discriminate between interactive and non-interactive pairs of host and viral proteins (62). Specifically, it tends to find the hyperplane that maximizes the margin i.e., the distance between data points of the corresponding classes. Furthermore, to handle non-linear feature space, the SVM classifier facilitates kernel trick where non-linear feature space is transformed to separable linear feature space. Considering the promising predictive performance of the SVM classifier in various proteomics sequence analysis tasks including coronavirus survival prediction (63), hepatitis B-related hepatocellular carcinoma recurrence prediction (64), sulfenylation sites prediction (65). As a whole, the SVM classifier has achieved an average performance of more than 80%. We have used an SVM classifier to distinguish interactive virus-host protein sequences from non-interactive ones.

#### 2.3.2. Random forest classifier

A random forest classifier is based on decision trees (DT), which are considered the base learners (66). To begin with, a root node is selected according to the feature with the lowest Gini impurity or the maximum information gain (67). Several samples are then separated based on the classes relevant to the selected feature. The process is repeated until all nodes are homogeneous or simply all nodes contain data only related to one class.

Random Forest classifier is a collection of hundreds of trees such that each tree is grown using a bootstrap sample of the original data (68). In a random forest each tree is grown in a nondeterministic way by inducing randomness at two different stages. First, randomness occurs at the tree level, as different trees get bootstraps of samples. At the node level, randomization is introduced by selecting a random subset of features for finding the best split rather than growing a tree on the complete feature set. This randomness helps in decorrelating the individual trees such that the whole forest has a low variance. Finally, an averaged or a voted decision is formulated based on individual predictions from the DTs. Following the success of the RF classifier in distinct proteomics sequence analysis tasks such as host disease classification (69), urine proteome profiling (70), and hydroxyproline and hydroxylysine site prediction (71). RF classifier manages to achieve an average performance of more than 80% on these tasks. We have utilized RF classifier to compute a discriminative and informative feature space using the separate statistical representations of APAAC and QS order sequence encoders as well as combined statistical representations.

#### 2.3.3. Extra trees classifier

Extremely randomized trees or extra trees (ET) classifier makes predictions similar to RF classifier (72). Multiple trees are trained and each tree is exposed to all training data. ET drops the idea of bootstrapping and takes a random subset of features without replacement. Unlike RF classifier, in the ET classifier, the splits are created across nodes *via* random splitting not based on the best splitting. Therefore, the ET classifier provides independent trees which yield better accuracy scores and low variance across different classes. Considering the increasing usage of ET classifiers in proteomics sequence analysis tasks including glutarylation sites prediction (73), non-coding RNA-protein interaction prediction (74), and protein stability changes estimation (75). Overall, the ET classifier marks an average performance of more than 80% on these tasks. We have used the ET classifier to assist the RF classifier in the generation of effective feature space using statistical representations of two physico-chemical properties based sequence encoders.

## 3. Evaluation criteria

To evaluate the integrity, effectiveness, and prediction performance of the proposed virus-host PPIs meta predictor in a reliable manner, following the evaluation criteria of existing studies (21, 30, 36), we utilize 8 different evaluation measures, i.e., accuracy (ACC), specificity (SP), sensitivity (SN), precision (PR), F1 score, area under the receiver operating characteristic (AUROC), area under the precision-recall curve (AUPRC) and Matthews correlation coefficient (MCC).

Accuracy (ACC) (30) measures the proportion of correct predictions with respect to total predictions. Specificity (SP) or True Negative Rate (TNR) is the ratio between true negative class predictions and overall predictions of negative class. Similarly, Recall/Sensitivity (21) computes the score by taking the ratio of correct predictions made on positive class samples to the sum of correct and false predictions of positive class samples. Precision (PR) computes performance score by taking the ratio between correct predictions of positive class samples and all samples which predictor labeled as the positive class. The area under receiver operating characteristics (AUROC) (32) calculates performance score by using true positive rate (TPR) and false positive rate (FPR) at different thresholds. Whereas, the area under the precision-recall curve (AUPRC) (32) calculates the performance scores using precision (P) and recall (FPR) at different thresholds. F1 score combines the precision (P) and recall (R) into a single measure by taking their harmonic mean. MCC (21) measures the correlation of the true classes with the predicted classes by considering all predictions related to positive and negative class samples. Mathematical equations of the aforementioned measures are given as,


(13) $$f(x)=\{\begin{array}{l} \text{ACC}=(T_{P}+T_{N})/(T_{P}+T_{N}+F_{P}+F_{N})\\ \text{Specificity(SP)}=T_{N}/(T_{N}+F_{P})\\ \text{Sensitivity(SN)orRecall(R)}=T_{P}/(T_{P}+F_{N})\\ \text{Precision(P)}=T_{P}/(T_{P}+F_{P})\\ \text{TruePositiveRate(TPR)}=T_{P}/(T_{P}+F_{N})\\ \text{FalsePositiveRate(FPR)}=F_{P}/(T_{N}+F_{P})\\ \text{F1score}=2(P\times R)/(P+R)\\ \text{MCC}=T_{P}\times T_{N}-F_{P}\times F_{N}/E\\ \text{E}=\sqrt{\left(T_{P}+F_{N})(T_{P}+F_{P})(T_{N}+F_{P})(T_{N}+F_{N}\right)} \end{array}$$

In the above numerical cases, *T*_*P*_ and *T*_*N*_ denote true predictions on the positive and negative classes. While *F*_*P*_ and *F*_*N*_ refer to the false predictions related to the positive and negative classes, respectively.

To compute the performance of the proposed predictor in terms of the aforementioned evaluation measures, the evaluation of the predictive pipeline can be performed under the hood of two different settings, k-fold cross-validation and independent test set based evaluation. In k-fold cross-validation, corpus sequences are split into k-folds where iteratively k-1 folds are used for training and the remaining fold is used for testing the predictive pipeline. In this setting, train test split biaseness does not exist as each sequence participates in training and evaluation. Whereas in the independent test set based evaluation, standard train and test splits of sequences are already available, hence training sequences are used to train the predictive pipeline and test sequences are used to test the predictive pipeline. There is a possibility that authors of benchmark datasets may partition hard sequences in the training set and simple sequences in the test set or vice versa. However, we believe that authors of benchmark datasets have carefully partitioned the sequences into train and test sets to avoid any biaseness and perform a fair evaluation. In our study, following previous work (22, 29–31) which has performed independent test set based evaluation on all datasets except Barman et al. (22) dataset, we also perform independent test set based evaluation to make a fair performance comparison. Contrarily, we evaluate our proposed predictor on Barman et al. (22) dataset using 5-fold cross-validation as done by the previous benchmark study.

## 4. Experimental setup

The proposed meta-predictor is implemented in Python and protein sequence encoders i.e., APAAC and Qsorder are taken from iLearnPlus (76). The classifiers are implemented using Scikit-Learn (77). In order to determine the optimal hyperparameters of encoding methods and classifiers, we have utilized a grid search strategy (78). A search in the grid finds the most optimal values of hyperparameters by evaluating all possible parameter combinations.

In order to determine the optimal parameters λ for the APAAC encoder and lag for the Qsorder encoder, *en*_λ, *lag*_ = [1, ⋯ , 5] is used as the grid search space with a stride size of 1. For the Qsorder encoder, lag=1 is chosen for Denovo, TR3-TS1, and Coronavirus datasets, lag=2 is selected for Barman, TR1-TS1 datasets, lag=3 for TR2-TS2, and lag=4 for TR4-TS4. In addition for the APAAC encoder, λ = (4, 4, 4, 4, 3, 3, 1), are chosen for Barman, Denovo, TR2-TS2, TR4-TS2, Coronavirus, TR1-TS1, and TR3-TS1 datasets.

In terms of ML classifiers, the performance of the SVM classifier is greatly influenced by the base kernel, along with the regularization parameter penalty *C* that controls the margin among hyperplanes. Whereas the parameter *d* represents the degree of the polynomial kernel, which affects the flexibility of the decision boundary in SVM. Similarly, the performance of tree-based classifiers (RF, ET) relies on the number of base estimators, maximum features for the best split, and splitting criteria i.e., Gini impurity and entropy. To make sure the reproducibility of results, the grid search space, and the selected hyperparameters are shown in Table 1.

**Table 1:** Grid search parameters along with optimal values for 3 different machine learning classifiers used for virus-host protein-protein interaction prediction.

	**Selected parameters**			**Grid search space**		
**Datasest**	**RF**	**ET**	**SVM**	**RF**	**ET**	**SVM**
Barman	(100, gini, auto)	(100, entropy)	(poly, 1, 100, 0.001, true)	*n*_*be*_=[30, 60, 100, 150, 200, 250, 300, 350, 400, 450, 500], c=[gini, entropy] max_*fea*_=[auto, *log*_2_, sqrt]	*n*_*be*_=[30, 60, 100, 150, 200, 250, 300, 350, 400, 450, 500], c=[gini, entropy]	K=[rbf, poly, linear, sigmoid], d=[1, 2, 3, 4, 5], C=[1, ⋯ , 100], γ=[auto, scale], prob=[True, False]
Denovo	(300, gini, null)	(300, gini)	(poly, 1, 100, 0.01, true)			
Coronavirus	(100, gini, auto)	(100, entropy)	(linear, 3, 5, 0.0001, true)			
TR1-TS1	(100, gini, auto)	(30, entropy)	(poly, 2, 100, 0.0001, true)			
TR2-TS2	(100, gini, auto)	(30, entropy)	(poly, 3, 10, 0.1, true)			
TR3-TS1	(100, gini, auto)	(30, entropy)	(poly, 2, 100, 0.001, true)			
TR4-TS2	(100, gini, auto)	(30, entropy)	(poly, 2, 100, 0.001, true)			

## 5. Results

This section briefly describes the performance of the proposed meta predictor at different levels of ensembling. Furthermore, it compares the performance of the proposed meta predictor with existing predictors (11, 21–23, 29, 30, 32, 36) over 7 different benchmark datasets (22, 29, 36, 43).

### 5.1. Performance analyzes of proposed meta predictor using different representations at property level and encoder level

The impact of different physicochemical properties and dimensionality reduction is explored by analyzing the performance of RF and SVM classifiers on the TR4-TS2 dataset. Table 2 shows 8 different evaluation measures based on performance values produced by the RF classifier using statistical representations generated through APAAC and Qsorder encoders using individual and combinations of properties. It also illustrates the performance values of the classifier using combined statistical vectors of both encoders. To illustrate the performance impact of dimensionality reduction, it shows the performance of the RF classifier using the feature agglomeration method based on generated comprehensive feature space of statistical vectors produced through individual encoders (APAAC, Qsorder) and a combination of both encoders. To illustrate, the performance gains achieved through iterative representation learning of second stage classifier using first stage classifier predicted probabilities, show the performance of the SVM classifier.

**Table 2:** Performance comparison of different statistical representations across 1st stage RF classifier and with iterative feature learning based 2nd stage SVM classifier.

**Encoder**	**Properties**	**DR**	**Random forest classifier**							
			**ACC**	**PR**	**F1**	**SP**	**SN**	**AUPRC**	**AUROC**	**MCC**
QSOrder	*p*_1_	no	84.16	85.46	84.01	84.06	91.01	98.02	97.55	69.91
	*p*_2_	no	83.89	85.75	83.68	83.89	90.23	98.16	97.74	69.62
	*p*_1_+ *p*_2_	no	84.23	85.99	84.03	84.23	91.77	**98.24**	**97.89**	70.20
	*p*_1_+ *p*_2_	yes	**85.23**	**86.72**	**85.08**	**85.23**	**92.30**	97.90	97.49	**71.94**
APAAC	*p*_1_	no	83.22	85.80	82.91	83.22	90.22	98.09	97.49	68.97
	*p*_2_	no	82.89	85.05	82.62	82.89	89.45	98.04	97.35	67.90
	*p*_3_	no	84.56	86.71	84.34	84.56	91.45	**98.30**	97.88	71.24
	*p*_3_+ *p*_1_	no	82.89	85.30	82.59	82.89	89.45	98.10	97.49	68.15
	*p*_3_+*p*_2_	no	85.57	87.65	85.37	85.57	92.59	98.24	97.75	73.19
	*p*_3_+*p*_2_	yes	**86.24**	**88.36**	**86.05**	**86.24**	**92.98**	98.26	**97.96**	**74.57**
	*p*_1_+*p*_2_+*p*_3_	no	85.23	87.17	85.04	85.23	92.23	98.16	97.63	72.38
**APAAC+QSorder**		no	86.24	87.88	86.09	86.24	92.90	98.10	97.49	74.10
		yes	86.24	87.88	86.09	86.24	92.91	**98.24**	**97.80**	74.10
**2***nd* **Stage predictors**			**SVM classifier**							
Qsorder-DR-RF, APAAC-DR-RF, Qsorder-DR+APAAC-DR-RF, Qsorder-DR-ET, APAAC-DR-ET, Qsorder-DR+APAAC-DR-ET			**93.62**	**93.64**	**93.62**	**93.62**	**96.71**	**98.50**	**98.14**	**87.27**

Bold values denote the highest performance figures.

In Table 2, for the Qsorder encoder, *p*_1_ represents the Schneider-Wrede property and *p*_2_ denotes the Grantham property. Similarly for the APAAC encoder, *p*_1_, *p*_2_, and *p*_3_ denote hydrophobicity, hydrophilicity, and side chain mass properties, respectively. RF classifier with statistical vectors generated through Qsorder using the *p*_1_ property produces 84.16% accuracy and 83.89% accuracy using the *p*_2_ property. It can be concluded that, RF classifier produces different performances when it is fed with two different statistical vectors generated through the Qsorder encoder by using two different physicochemical properties *p*_1_ and *p*_2_. This performance difference illustrates both properties extract and encode different types of information while generating statistical vectors. The performance of the classifier is improved when it is fed with combined statistical vectors generated through both properties. Its performance gets further improved when it is fed with combined vectors of both properties reduced through the feature agglomeration method. This performance improvement validates, that both properties extract some redundant features that when eradicated in the newly generated feature space, the performance gets improved.

Similarly, for the APAAC encoder, among 3 statistical vectors generated through 3 different properties, the RF classifier produces better performance with the *p*_3_ property and produces the lowest performance with the *p*_2_ property. Thus, according to the working paradigm of the proposed property selection method, top-performing property *p*_3_ vectors will combine with *p*_1_ and *p*_2_ properties vectors iteratively. From the concatenation of the *p*_3_ property vector with *p*_1_ and *p*_2_ property vectors, the classifier achieves a slight performance gain with *p*_3_ and *p*_2_ concatenation. Furthermore, when *p*_3_ and *p*_2_ properties vectors were combined with the *p*_1_ property, the performance of the classifier decreased as compared to its performance with *p*_2_ and *p*_3_ properties combinations, and the property selection method selected *p*_2_ and *p*_3_ as two optimal properties. These results reveal that to fully utilize the potential of the APAAC encoder, it is essential to utilize the best combination of properties. Furthermore, the concatenation of statistical vectors generated through selected best properties based APAAC and Qsorder encoders fails to improve the performance of the RF classifier as compared to its performance on individual statistical representations.

Dimensionality reduction along with individual encoders has improved the performance of the RF classifier as compared to its performance on the same encoders without applying dimensionality reduction. However, it produces almost similar performance with and without dimensionality reduction on combined vectors of APAAC and Qsorder encoders.

To gain further performance enhancement, in the second stage we utilize positive class probabilities predicted by ET and RF classifiers using feature agglomeration based optimized statistical vectors of individual APAAC and Qsorder encoders and both encoders combined vectors. SVM classifier is trained on newly generated probabilistic 6D feature space where it achieves higher performance as compared to the performance values of RF and ET classifiers. In comparison to the performance of the RF classifier with sequence representations obtained by applying dimensionality reduction to APAAC and QS Order combined vectors (APAAC+Qsorder, DR=yes), it achieves performance improvements of 7.38% in accuracy, 5.76% in precision, 7.53% in F1 score, 7.38% in specificity, 3.1% in sensitivity, 0.26% in AUPRC, 0.34% in AUROC and 13.17% in MCC. In comparison to the performance of the RF classifier with sequence representations generated through (*p*_1_+*p*_2_, DR=yes) of Qsorder and (*p*_3_+*p*_2_, DR=yes) of APAAC, it achieves performance improvements with an average margin of 6.10% across all the evaluation measures. Therefore, it is inferred that the SVM classifier along with the iterative representation learning leads to the highest performance for virus-host protein-protein interaction prediction.

#### 5.1.1. Proposed MP-VHPPI predictor performance comparison with existing predictors on Barman's dataset

Table 3 shows the performance values of 7 different evaluation measures of the proposed meta predictor and 6 existing VHPPI predictors (22, 30–32, 38) on Barman et al.'s (22) dataset. From 6 existing predictors, Asim et al. (38) LGCA-VHPPI predictor achieves better performance in terms of accuracy 82%, specificity 89.37%, f1 score 81.47%, MCC 63.99%, and AU-ROC 88%. Whereas, Zhou et al., (30) predictor produce better performance in terms of precision at 82.46%. Among 7 different evaluation measures, Barman et al., predictor (22) only managed to produce the highest sensitivity 89.08% as compared to the sensitivity of 5 other predictors. Comparatively, the proposed meta-predictor outperforms 6 previously mentioned predictors (22, 30–32, 38) in terms of 6 distinct evaluation measures. Overall, in terms of accuracy, the proposed meta predictor achieves an improvement of 0.9%, 1.79% in sensitivity 1.62% increase in precision, 1.27% increase in F1 score, 2.97% in MCC, and 0.17% in terms of AU-ROC.

**Table 3:** Performance comparison of proposed MP-VHPPI with existing viral-host PPI predictors over a benchmark Barman dataset in terms of 7 different evaluation measures.

**Approach**	**ACC**	**SN**	**SP**	**PR**	**F1**	**MCC**	**AU-ROC**
Yang et al. (31) RF	79.17	81.85	76.45	77.83	79.79	58.40	87.1
Alguwzizani et al. (32) SVM	78.6	73.72	83.48	81.69	77.50	57.50	84.70
Barman et al. (22) SVM	71.00	67.00	74.00	72.00	69.41	44.0	73.00
Barman et al. (22) RF	72.41	89.08	55.66	82.26	66.39	48.00	76.00
Zhou et al. (30) SVM	79.95	76.14	83.77	82.46	79.17	60.1	85.8
Asim et al. (38) LGCA-VHPPI	82.00	82.00	**89.37**	82.40	81.47	63.99	88.00
**Proposed MP-VHPPI**	**82.90**	**90.87**	82.90	**84.08**	**82.74**	**66.96**	**88.17**

Performance figures of Barman et al. (22) SVM, Barman et al. (22) RF, Alguwzizani et al. (32) SVM, and Yang et al. (31) RF are taken from Yang et al. (31) work. Bold values denote the highest performance figures.

In terms of robustness on Barman's dataset, the proposed and existing predictors fall into two different categories based on the differences between their specificity and sensitivity scores, i.e., less biased, predictors with a small difference in specificity and sensitivity scores, and more biased predictors with a large difference in specificity and sensitivity scores. Individually there are sensitivity and specificity differences of 5.4, 9.76, 7, 33.42, 7.63, 7.37, and 7.97% for Yang et al.'s (31) RF, Alguwzizani et al. (32) SVM, Barman et al. (22) SVM, and Barman et al. (22) RF, Zhou et al. (30) SVM, Asim et al. (38) LGCA-VHPPI predictor and the proposed meta-predictor, respectively. On the basis of these difference values, among all predictors, Yang et al.'s RF (31), Barman et al. (22) SVM, Zhou et al. (30) SVM, Asim et al. (38) LGCA-VHPPI, and proposed meta-predictor can be considered less biased as they have small differences (< 8%) in terms of their specificity and sensitivity scores. Contrarily, the other two predictors Barman et al.'s (22) RF, and Alguwzizani et al. (32) SVM, have large differences between sensitivity and specificity scores and are biased toward either type I or type II error. Type I error arises when a predictor is prone toward false positive predictions due to low specificity and high sensitivity scores (*T*_*I*_*E* = 1−*SP*), and in type II error the predictor is prone to false negative predictions due to low sensitivity and high specificity scores (*T*_*II*_*E* = 1−*SN*). Barman et al.'s (22) RF is more prone to type I error due to high sensitivity and lower specificity scores, whereas Alguwzizani et al. (32) SVM is more prone to type II error due to higher specificity and lower sensitivity scores.

#### 5.1.2. Proposed MP-VHPPI predictor performance comparison with existing predictors on Denovo's dataset

Table 4 illustrates performance values of 7 different evaluation measures of the proposed meta predictor and 7 existing VHPPI predictors Yang et al. (31) RF, Alguwzizani et al. (32) SVM, Fatma et al. (29) SVM, Yang et al. (36) CNN, Zhou et al. (30) SVM, Dong et al. (23) LSTM, and Asim et al. (29) LCGA-VHPPI on Denovo dataset.

**Table 4:** Performance comparison of proposed MP-VHPPI with existing viral-host PPI predictors over benchmark DeNovo dataset (29) in terms of 7 different evaluation measures.

**Approach**	**ACC**	**SN**	**SP**	**PR**	**F1**	**MCC**	**AU-ROC**
Yang et al. (31) RF	93.23	90.33	96.17	95.99	93.07	86.60	98.10
Alguwzizani et al. (32) SVM	86.47	86.35	86.59	86.56	86.46	72.90	92.60
Fatma et al. (29) SVM	81.90	80.71	83.06	–	–	–	–
Yang et al. (36) CNN	94.12	90.82	**97.41**	**97.23**	93.92	–	–
Zhou et al. (30) SVM	84.47	80.00	88.94	87.86	–	62.92	89.7
Dong et al. (23) LSTM	–	84.12	–	83.92	84.02	–	92.21
Asim et al. (38) LGCA-VHPPI	94.24	94.24	96.47	94.32	94.23	88.56	**98.49**
**Proposed MP-VHPPI**	**94.59**	**97.23**	94.59	94.73	**94.58**	**89.32**	98.16

Performance figures of DeNovo SVM (29), Alguwzizani et al. (32) SVM, and Yang et al. RF on DeNovo Dataset (29) are taken from Yang et al. (31) work. Bold values denote the highest performance figures.

From 7 existing predictors, Asim et al., LGCA-VHPPI predictor (38) achieve better performance in terms of accuracy 94.24%, sensitivity 94.24%, f1 score 94.23%, MCC 88.56%, and AU-ROC 98.49%. Whereas, Yang et al., predictor (36) achieve the highest performance values in terms of specificity 97.41%, and precision 97.23%. Among all existing predictors, Fatma et al., predictor (29) show the least performance. In comparison to these predictors, the proposed meta predictor offers performance improvements across 4 different evaluation measures. It achieves a performance gain of 0.35% in both accuracy and f1 score, 2.99% increment in sensitivity, and 0.76% increment in MCC.

The predictors on the Denovo dataset can be seen in two different categories as done previously in terms of Barman's dataset on the basis of specificity and sensitivity differences. Individually there exist differences of 5.84, 6.59, 2.35, 2.23, and 2.64% across Yang et al. (31) predictor, Yang et al. (36) CNN, Fatma et al. (29), Asim et al. (38) LGCA-VHPPI, and proposed meta-predictor. Due to less difference (< 3%) in the specificity and sensitivity scores, Alguwzizani et al. (32), Fatma et al. (29), Asim et al. (38) LGCA-VHPPI, and the proposed meta-predictor can be considered less biased toward type I and type II errors as compared to the other two predictors i.e., Yang et al. (31) RF and Yang et al. (36) CNN that are more biased toward type II error due to high specificity and low sensitivity scores.

#### 5.1.3. Proposed MP-VHPPI predictor performance comparison with existing predictors on coronavirus dataset

Due to the recent pandemic of Coronavirus, it is important to analyze the performance of a predictor on Coronavirus and human proteins. Table 5 shows the performance values of the proposed meta predictor, Yang et al. (36) CNN, and Asim et al. (38) LGCA-VHPPI, across Coronavirus and human proteins dataset (36), in terms of 8 distinct evaluation measures.

**Table 5:** Performance comparison of the proposed predictor with the existing Yang et al. (36) predictor over the Coronavirus dataset.

**Approach**	**ACC**	**SN**	**SP**	**PR**	**F1**	**MCC**	**AU-PRC**	**AU-ROC**
Yang et al. (36) CNN	90.64	16.37	**98.06**	45.81	24.12	-	32.9	-
Asim et al. (38) LGCA-VHPPI	90.11	93.6	50.04	85.67	85.07	**22.21**	38.01	80.0
**Proposed MP-VHPPI**	**91.18**	**95.58**	51.74	**86.01**	**87.27**	10.08	**47.07**	**82.95**

Bold values denote the highest performance figures.

Out of two existing predictors, Yang et al., a predictor based on CNN achieve better accuracy 90.64%. Whereas, Asim et al. (38) LGCA-VHPPI predictor shows better performance in terms of, sensitivity of 93.6%, precision of 85.67%, AU-PRC 38.01%, and f1 score of 85.07%. Due to the highly imbalanced number of samples for interactive and non-interactive classes in the Coronavirus dataset, Yang et al. (36) predictor perform poorly as evidenced by its extremely low sensitivity, precision, F1, and AU-PRC scores. The proposed meta predictor outperforms existing predictors in terms of accuracy by a margin of 0.54%, 1.98% in sensitivity, 0.34% in precision, 2.2% in f1 score, 9.06% in terms of AU-PRC, and 2.95% in AU-ROC. Only in terms of MCC, Asim et al., LGCA-VHPPI predictor (38) perform better than the proposed meta predictor by achieving an increment of 12%.

Individually, there exist differences of 81.69, 43.56, and 43.84% in specificity and sensitivity scores for Yang et al. (36) predictor, Asim et al. (38) LGCA-VHPPI predictor, and the proposed meta predictor. On the basis of that, it can be inferred that the proposed meta-predictor and Asim et al. (38) LGCA-VHPPI predictor are less biased toward type I and type II errors. Whereas, Yang et al. (36) predictor is biased toward type II error due to high specificity and low sensitivity scores.

### 5.2. Proposed predictor performance comparison with existing predictors on unseen viruses test sets

To assess the applicability of the VHPPI predictors on unseen viruses where predictors are trained on different types of viruses and evaluation is performed on the test sets that contain viruses (Influenza A virus subtype H1N1, and Ebola virus EBV) that are not part of the training sets. Table 6 compares the performance values of the proposed meta-predictor with 4 existing predictors i.e., Zhou et al. (30) SVM, Tsukiyama et al. (21) LSTM-PHV, Dong et al. (23) predictor, and Asim et al. (38) LGCA-VHPPI.

**Table 6:** Performance comparison of the proposed MP-VHPPI with existing virus-Host PPI Predictors over 4 datasets developed by Zhou et al. (30), to assess the applicability of the unseen viruses.

**Dataset**	**Approach**	**ACC**	**SN**	**SP**	**PR**	**F1**	**MCC**	**AU-ROC**
**TR1-TS1**	Zhou et al. (30) (SVM)	77.95	89.76	66.14	72.61	-	57.5	88.6
	Tsukiyama el al. (21) LSTM-PHV	86.7	90.6	82.9	84.1	-	73.7	91.2
	Dong et al. (23) LSTM	–	86.51	–	86.28	86.40	–	94.61
	Asim et al. (38) LGCA-VHPPI	83.82	91.48	83.82	85.34	83.64	69.14	94.0
	**Proposed MP-VHPPI**	**90.26**	**95.06**	**90.26**	**91.44**	**90.19**	**81.69**	**96.70**
**TR2-TS2**	Zhou et al. (30)(SVM)	78.00	90.67	65.33	72.34	-	57.9	86.7
	Tsukiyama el. al. (21) LSTM-PHV	84.0	93.3	74.7	78.7	-	69.2	94.1
	Dong et al. (23) LSTM	–	92.53	–	90.93	91.23	–	96.80
	Asim et al. (38) LGCA-VHPPI	86.58	93.11	86.57	88.35	86.42	74.9	96.0
	**Proposed MP-VHPPI**	**94.30**	**97.07**	**94.30**	**94.39**	**94.29**	**88.69**	**97.77**
**TR3-TS1**	Zhou et al. (30) (SVM)	77.43	88.98	65.88	72.28	-	56.4	88.4
	Tsukiyama el al. (21) LSTM-PHV	85.7	89.2	82.2	83.3	-	71.6	92.1
	Asim et al. (38) LGCA-VHPPI	83.29	91.2	83.28	85.31	83.05	68.57	94.0
	**Proposed MP-VHPPI**	**90.53**	**95.06**	**90.53**	**90.78**	**90.51**	**81.31**	**95.98**
**TR4-TS2**	Zhou et al. (30) (SVM)	81.67	94.67	68.67	75.13	-	65.6	89.0
	Tsukiyama el al. (21) LSTM-PHV	90.0	91.3	88.7	89.0	-	80.0	95.6
	Asim et al. (38) LGCA-VHPPI	85.57	92.59	85.57	87.65	85.37	73.19	96.0
	**Proposed MP-VHPPI**	**93.62**	**96.71**	**93.62**	**93.64**	**93.62**	**87.27**	**98.14**

The performance values of the existing predictors i.e., Zhou et al. (30) and Tsukiyama et al. (21) (LSTM-PHV) are taken from their corresponding studies (21, 30).

Over the TR1-TS1 dataset, out of 4 existing predictors Tsukiyama et al. (21) LSTM-PHV performs better in terms of accuracy 86.7% and MCC 73.7%, Dong et al. (23) predictor shows the highest precision 86.28%, f1 score 86.40%, and AUROC 94.61%. Asim et al., LCGA-VHPPI shows the highest performance in terms of specificity and sensitivity i.e., 83.82 and 91.48%. Whereas, Zhou et al. (30) predictor show the least performance across all evaluation measures except sensitivity. In comparison to the existing predictors, the proposed meta predictor outperforms existing predictors across 7 evaluation measures. It achieves an increase of 3.56% in accuracy, 6.44% in specificity, 3.58% in sensitivity, 5.16% in precision, 3.79% in F1 score, 7.99% in MCC, and 2.09% in AUROC. Three out of 4 existing predictors, Tsukiyama et al. (21) LSTM-PHV, Zhou et al. (30) SVM, and Asim et al. (38) LGCA-VHPPI, are biased toward type 1 error due to lower specificity (82.9, 66.14, and 83.82%) and higher sensitivity scores (90.6, 89.76, and 91.48%) with differences of 7.7, 23.62, and 7.66%. In comparison, the proposed meta predictor is robust and generalizable due to the small difference between specificity and sensitivity scores i.e., 4.8%, and overall higher sensitivity, specificity, AUROC, accuracy, and MCC scores.

Over the TR2-TS2 dataset, out of four existing predictors Tsukiyama et al., LSTM-PHV performs better in terms of sensitivity 93.3%, whereas Dong et al. (23) predictor performs better in terms of precision 90.93%, f1 score 91.23%, and AUROC 96.80%. Asim et al. (38) LGCA-VHPPI predictor performs better in terms of accuracy 86.58%, specificity 86.57%, and MCC 74.9%. Zhou et al. (30) predictor, shows the least performance across all the evaluation metrics except sensitivity at 90.67%. The proposed meta predictor outperforms existing predictors across all of the evaluation measures. Overall, the proposed meta predictor achieves a gain of 7.72% in accuracy, 3.77% increase in sensitivity, 7.73% in specificity, 3.46% in precision, 3.06% in F1, 13.79% in MCC, and 0.97% in AUROC. Among these predictors, the predictors of Tsukiyama (21), Zhou et al. (30), and Asim et al. (38) LGCA-VHPPI, are prone to type 1 error due to high sensitivity and low specificity scores. For instance, the difference in specificity and sensitivity scores of Zhou et al. predictor is 25.34%, 18.6% for Tsukiyama et al. (21) LSTM-PHV, and 6.54% for Asim et al. (38) LGCA-VHPPI predictor. Due to these big differences, these predictors do not generalize well against the human and Ebola virus protein data. Whereas, the proposed meta-predictor has a smaller difference of 2.77% between specificity and sensitivity values, which makes it more generalizable than existing predictors.

Out of three existing predictors, the LSTM-PHV predictor performs better across TR3-TS1 in terms of 2 different evaluation metrics i.e., 85.7%, 71.6%, for accuracy, and MCC. Similarly, Asim et al. (38) LGCA-VHPPI shows better performance in terms of sensitivity 91.2%, specificity 83.28%, precision 85.31%, and AU-ROC 94.0%. On the other hand, the proposed meta predictor outperforms existing predictors on 7 different evaluation measures by significant margins. The proposed meta predictor achieves a raise of 4.83% in accuracy, 3.86% in sensitivity, 7.25% in specificity, 5.47% in precision, 9.71% in MCC, 7.46% in f1, and 1.98% in AUROC. Similar to the previous cases, existing predictors are again prone to type 1 errors due to high sensitivity and low specificity scores with differences of 23.1, 7, and 7.92% for Zhou et al. (30), LSTM-PHV (21), and LGCA-VHPPI (38) predictors. Comparatively, the proposed meta-predictor has a smaller difference of 4.53% between specificity and sensitivity scores, which makes the proposed meta-predictor more suitable for VHPPI prediction.

Over the TR4-TS2 dataset out of three existing predictors, LSTM-PHV (21) achieves better results across 4 evaluation measures i.e., 90.0%, 88.7%, 89.0%, 80.0%, in terms of accuracy, specificity, precision, and MCC. LGCA-VHPPI (38) excels in terms of AU-ROC 96.0%. Whereas, Zhou et al. (30) SVM shows a better sensitivity score of 94.67%. The proposed predictor achieves performance gains of 3.62% in accuracy, 2.04% in sensitivity, 4.92% in specificity, 4.64% in precision, 8.25% in f1 score, 7.27% in MCC and 2.14% in AU-ROC. There exists a difference in the specificity and sensitivity scores of these predictors which are 26% for Zhou et al. predictor and 7.02% for Asim et al. (38) LGCA-VHPPI, which makes them more biased toward type I error due to high sensitivity and lower specificity scores. Comparatively, LSTM-PHV and the proposed meta predictor have a lower difference in specificity and sensitivity scores of (< 3.1%), which suggests that for the TR4-TS2 dataset, both of the predictors are able to generalize well over positive and negative class samples.

## 6. Discussion

Since the last decade, the development of machine and deep learning-based computational approaches for virus-host protein-protein interaction prediction has been an active area of research (21, 22). In the marathon of developing robust computational VHPPI predictors, the aim of each newly developed predictor has been to utilize raw virus-host protein sequences and precisely discriminate interactive viral-host protein sequences from non-interactive ones. However, most predictors have been evaluated on a limited type of viruses and hosts, such as 6 different predictors have been evaluated on the Barman dataset that contains 5 different viruses and human proteins as hosts. Seven predictors are evaluated on the Denovo dataset which is comprised of 10 different viruses and human proteins as hosts, and 2 predictors are evaluated on the Coronavirus virus. Only 4 predictors are evaluated on the Zhou et al. (30) dataset, which consists of 332 viruses and 29 host proteins. These datasets are more suitable to evaluate the robustness, generalizability, and predictive performance of a computational predictor. These datasets were developed with the objective to train models on different types of viruses and evaluate them on the particular viruses which were not part of the training set.

Over unseen virus-host protein-protein interaction prediction datasets, the performance of existing predictors is comparably low, as compared to their performance on Barman and Denovo datasets. Recently, we developed a machine learning-based predictor namely LGCA-VHPPI (38), which produced state-of-the-art performance on both Barman and Denovo datasets. We evaluated our predictor on Zhou et al. (30) datasets, where it showed a relatively lower performance as compared to its performance on Barman and Denovo datasets. This motivated us to develop an improved predictor that makes the best use of raw viral host protein sequences to perform better not only on Barman and Denovo datasets but also produce a similar performance for unseen viral-host protein-protein interaction predictions.

**Figure 3:**
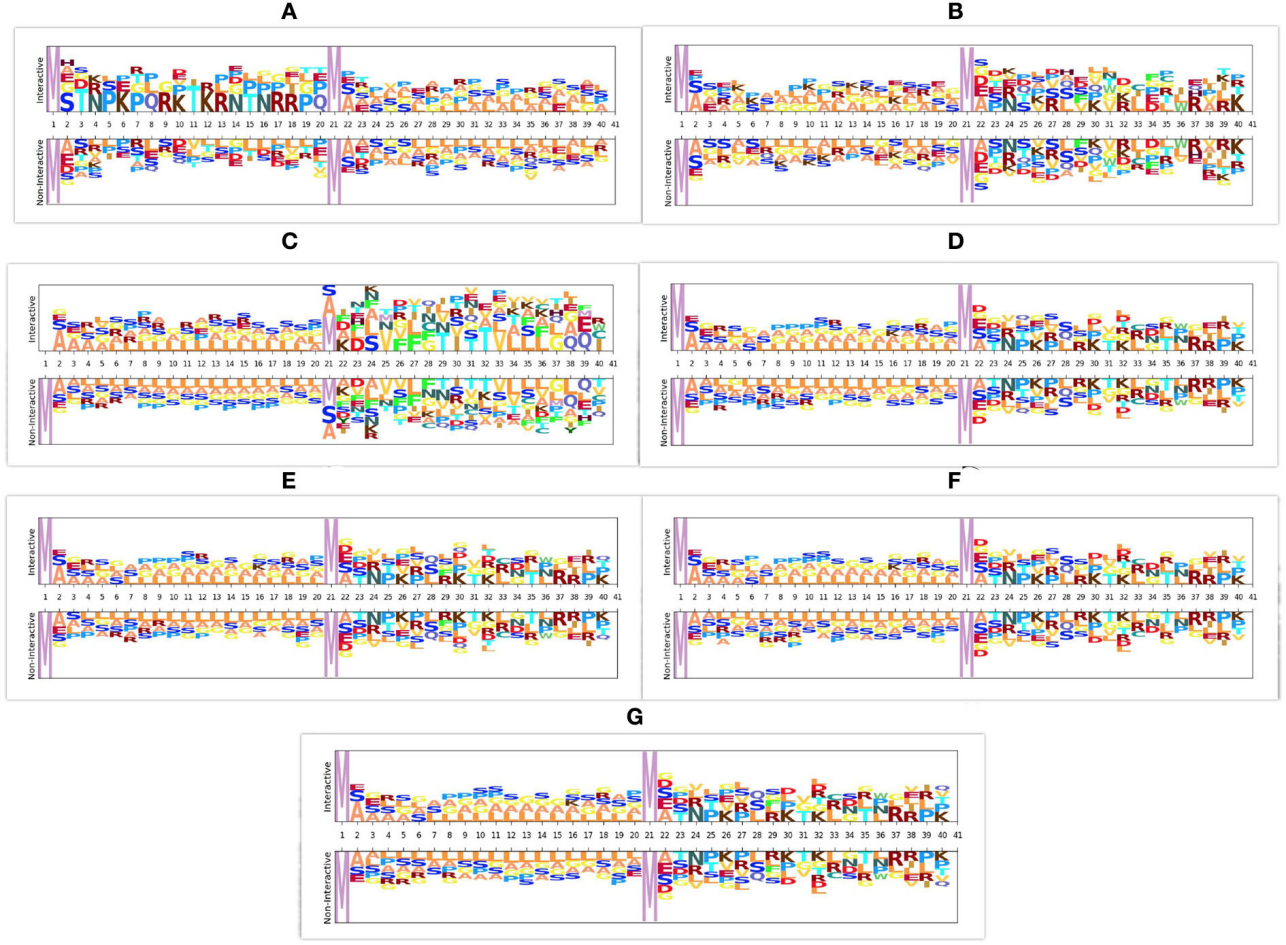
Distribution of amino acid sequences across 7 different datasets. For each dataset the distribution of amino acids is shown across interactive and non-interactive protein samples, **(A)** Barman dataset **(B)** Denovo dataset **(C)** Coronavirus dataset **(D)** TR1-TS1 dataset **(E)** TR2-TS2 dataset **(F)** TR3-TS1 dataset **(G)** TR4-TS2 dataset.

In viral and host protein sequences, the distribution of amino acids is almost similar across interactive and non-interactive classes. However, an amino acid occurrence at the same position across the sequences of interactive and non-interactive classes varies. The prime reason behind the biaseness of existing viral-host protein-protein interaction predictors toward type I or type II errors is their inability to capture position specific discriminative distribution of amino acids across both classes. To better illustrate this phenomenon, we perform amino acid distribution analysis across both classes with the help of Two Sample Logo (79). Virus and host protein sequences are huge in length and visualizing the amino acid distribution across entire sequence lengths is not feasible at all. Hence, for the purpose of visualization, we take 20 amino acids from the start of virus protein sequences and 20 amino acids from the start of host protein sequences. Using reduced 40 amino acids based sub-sequences, Figure 3 illustrates the amino acid distribution across interactive and non-interactive classes for 7 different datasets. It can be seen that the distribution of amino acids is approximately similar in interactive and non-interactive classes. Considering Barman's dataset as an example (Figure 3A), in interactive and non-interactive samples, there are overlapping amino acids at every position i.e., for position 2, interactive samples contain one of the following amino acid, H, A, E, G, S whereas, non-interactive samples also contain one of the following amino acid, A, E, G, S. In both classes occurrence of 4 amino acids is the same while few samples of the interactive class contain amino acid H, a similar trend exists at other locations as well. Furthermore, other datasets also contain a similar distribution of amino acids as in the Barman dataset. It can be concluded that, across all 7 datasets, we observe the limited discriminative distribution of amino acids and because of that existing predictors lack in performance due to the utilization of sub-optimal sequence encoding methods that generate statistical vectors by neglecting most of the discriminative features about the distribution of amino acids in interactive and non-interactive classes. It is noteworthy to mention that the prime goal of visualizing the amino acid distribution across both classes is to demonstrate the significance of most effectively characterizing viral host protein sequences. However, we have taken entire viral-host protein sequences in our study to generate statistical vectors using physico-chemical properties based sequence encoders, informative feature space using dimensionality reduction algorithm, discriminative feature space using tree based classifiers, and final prediction using SVM classifier. A comprehensive performance comparison of the proposed predictor with VHPPI predictors proves that the proposed predictor manages to capture position specific discriminative distribution of amino acids across both classes.

Furthermore, it is important to mention that all the amino acids are either polar or non-polar in nature and can carry charges, such as out of 21 unique amino acids, 11 amino acids are polar in nature, 4 AAs carry a positive charge (R, D, H, K), 2 AAs carry a negative charge (D, E), and 5 AAs are neutral (C, Q, S, T, Y). Whereas, 10 amino acids are non-polar in nature (A, G, I, L, M, F, P, W, Y, V). Irrespective of positions aware occurrences, considering the overall distribution of amino acids in the protein sequence, charges can be computed by utilizing the physicochemical properties. Overall charge information of amino acids along with their distribution information can extract and encode more discriminative patterns.

**Figure 4:**
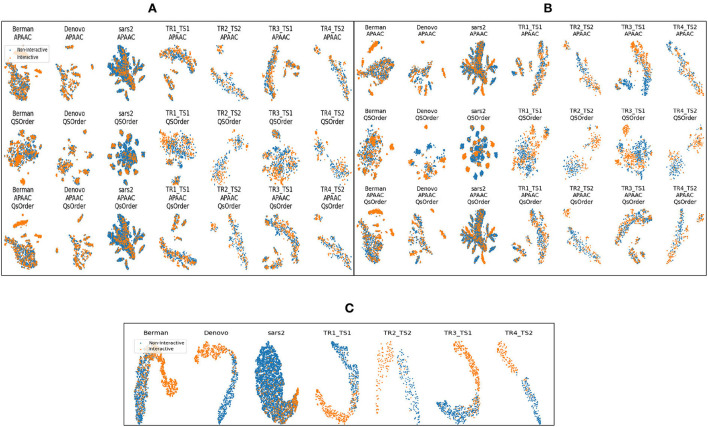
Formation of clusters with representations of protein sequences based on **(A)** APAAC and Qsorder encoders without dimensionality reduction **(B)** APAAC and Qsorder encoders with dimensionality reduction **(C)** the positive class probabilities through ET and RF classifiers.

Figure 4 shows different clusters of 7 benchmark datasets for the intrinsic analyzes of the statistical vectors generated through APAAC and Qsorder sequence encoders. These clusters are computed by first reducing the dimensions of statistical vectors through principal component analysis (PCA) and then by t-distributed stochastic neighbor embedding (TSNE). In Figures 4A,B, rows represent clusters of interactive and non-interactive classes based on statistical vectors generated through individual encoders (APAAC, Qsorder), and a combination of both encoders. Whereas, the columns represent 7 different benchmark datasets namely, Barman, Denovo, Coronavirus. TR1-TS1, TR2-TS2, TR3-TS1, and TR4-TS2. Overall, statistical vectors from APAAC and Qsorder without dimensionality reduction lead to the formation of overlapping clusters for interactive and non-interactive classes. This overlapping reveals that generated statistical vectors are almost similar and contain less discriminative information about interactive and non-interactive classes, as shown in Figure 4A. Furthermore, this overlapping behavior among clusters exists due to the extraction of some irrelevant and redundant features by different physicochemical properties. To eradicate such type of information, we utilize the feature agglomeration method with the objective to transform generated statistical vectors into a more informative and discriminative feature space. Comparatively, statistical representations of APAAC and Qsorder with dimensionality reduction lead to the formation of slightly unique yet heavily dependent clusters as shown in Figure 4B. Though these encodings could be used for classification purposes, however, still the performance would not be very promising. In addition, the clusters do not seem independent because a single human protein that interacts with some viral proteins, might not interact with some other viral proteins. This means that positive and negative class samples can have very similar representations due to the presence of such proteins. Although dimensionality reduction produces better feature space, however still clusters are not very much separable. To further improve the performance of the predictor, we perform iterative representation learning, where we pass 3 different statistical representations separately to RF and ET classifiers and take their predicted class probabilities to develop a new feature space. The generated feature space leads to the formation of unique and independent clusters as shown in Figure 4C, which suggests the presence of comprehensive discriminatory features for interactive and non-interactive VHPPI pairs. Due to the discriminative and informative nature of the newly generated feature space, we utilize this feature space to train the SVM classifier for virus-host protein-protein interaction prediction.

Overall, as compared to state-of-the-art predictors, the proposed predictor has shown a slight performance improvement on Barman and Denovo datasets, and significant performance improvements on SARS-CoV-2 datasets and the other 4 datasets namely, TR1-TS1, TR2-TS2, TR3-TS1, and TR4-TS2. We believe that the performance of the proposed predictor can be further improved by incorporating representations learned through diverse types of language models such as BERT, and XLNET.

## 7. Web-server

To facilitate the biological community, we provide an interactive, and user-friendly web server for the proposed meta-predictor which is available at https://sds_genetic_analysis.opendfki.de/MP-VHPPI/. This web server can be used to predict virus-host protein-protein interactions across 7 different datasets by using raw human and virus protein sequences. In addition, the online web server allows the user to train the proposed meta-predictor from scratch on all of the datastets. Moreover, it also provides 7 different benchmark datasets that are used in this study.

## 8. Conclusion

The prime objective of this research is the development of a robust machine learning-based computational framework capable of precisely predicting viral-host protein-protein interactions across a wide range of hosts and viruses. The proposed meta predictor makes use of APAAC and QS order sequence encoders for statistical representation generation and feature agglomeration method to refine feature space. Furthermore, the meta predictor utilizes the predictions of random forest and extra tree classifiers to feed the SVM classifier that makes final predictions. Experimental results reveal the competence of APAAC and QS order encoders for most effectively generating numerical representations of sequences by capturing amino acids sequence order and distributional information. We have observed dimensionality reduction method removes irrelevant and redundant information which slightly improves the performance of classifiers. The process of iterative representation learning in which predictions of RF and ET classifiers are passed to the SVM classifier significantly improves the accuracy of interaction predictions. The proposed meta predictor has evaluated over 7 benchmark datasets where it outperforms existing predictors with a significant margin of 3.07, 6.07, 2.95, and 2.85%, in terms of accuracy, MCC, precision, and sensitivity, respectively. We believe that the deployment of the proposed meta-predictor as a web interface will assist researchers and practitioners in analyzing the complex phenomenon of VHPPIs at a larger scale to unravel substantial drug targets and optimize antiviral strategies.

## 9. Limitations

Comprehensive performance analysis reveals that the proposed MP-VHPPI predictor manages to outperform existing viral-host protein-protein interaction predictors across 7 benchmark datasets by a decent margin. Although the use of different strategies at the level of representation learning reduces the prediction error decently, however, the proposed model lacks robustness as it is biased toward type II error. In the future, we will optimize the predictive pipeline of the proposed MP-VHPPI predictor with an aim to enhance robustness.

## Data availability statement

The benchmark datasets for this study can be found at: https://sds_genetic_analysis.opendfki.de/MP-VHPPI/Download/.

## Author contributions

MA conceptualized the presented idea and prepared graphics and developed web server. MA and MI performed data curation, formal analysis, validation, and investigation. MA and AF prepared the original draft and final manuscript under the supervision of AD and SA. All authors contributed to the article and approved the submitted version.

## Funding

This study was supported by Sartorius Artificial Intelligence Lab.

## Conflict of interest

The authors declare that the research was conducted in the absence of any commercial or financial relationships that could be construed as a potential conflict of interest.

## Publisher's note

All claims expressed in this article are solely those of the authors and do not necessarily represent those of their affiliated organizations, or those of the publisher, the editors and the reviewers. Any product that may be evaluated in this article, or claim that may be made by its manufacturer, is not guaranteed or endorsed by the publisher.
