# Supplementary material for: MP-VHPPI: Meta predictor for viral host protein-protein interaction prediction in multiple hosts and viruses
Source: Front Med (Lausanne). 2022 Nov 16;9:1025887. doi: 10.3389/fmed.2022.1025887 (PMC9709337; doi:10.3389/fmed.2022.1025887)
Supplement: Supplementary file 1 [file Data_Sheet_1.PDF]

Name	Dataset	Encoding Method	Classifiers	Reference	Advantages/ Disadvantages of Encoding Method
**Deep Learning Based Models**					
X.Yang et, al, 2021	**Viruses:** HIV, Herpes, Papiloma, Influenza, Hepatitis, Dengue, Zika, SARS-CoV-2<br><br>**Host:** Human	Position Specific Scoring Matrix (PSSM)	Siamese convolutional neural network (CNN)	Yang, X., Yang, S., Lian, X., Wuchty, S., & Zhang, Z. (2021). Transfer learning via multi-scale convolutional neural layers for human–virus protein–protein interaction prediction. *Bioinformatics*, *37*(24), 4771-4778.	- has an extensive information about the position of nucleotides.<br><br>-
Deep Viral, 2021	**Viruses:** Arenaviridae, Parvoviridae, Floviviridae, Orthomyxoviridae, Pneumonoviridae, Paramyxoviridae, Retroviridae, Herpesviridae, Polyomaviridae, Adenoviridae, Flaviviridae, Poxviridae, Hepadnaviridae, Papilomaviridae<br><br>**Host:** Human	DL2vec to generate ontologies and Word2vec for embeddings	CNN	Liu-Wei, W., Kafkas, Ş., Chen, J., Dimonaco, N. J., Tegnér, J., & Hoehndorf, R. (2021). DeepViral: prediction of novel virus–host interactions from protein sequences and infectious disease phenotypes. *Bioinformatics*, *37*(17), 2722-2729.	- Combining structural and content information together.
LSTM-PHV, 2021	**Virus:** SARS-CoV-2<br><br>**Host:** Human	Word2vec	LSTM	Tsukiyama, S., Hasan, M. M., Fujii, S., & Kurata, H. (2021). LSTM-PHV: prediction of human-virus protein–protein interactions by LSTM with word2vec. *Briefings in bioinformatics*, *22*(6), bbab228.	
Lei Ding, 2020	**Viruses:** 137 different types of viruses, Human SARS coronavirus, Ebola virus, Dengue virus, etc<br><br>**Hosts:** Homo sapiens , Mus musculus, Bos taurus, Rattus norvegicus, Non human Sus scrofa, Gallus gallus, Equus caballus, Drosophila melanogaster, Canis lupus familiaris, Arabidopsis thaliana, Escherichia coli, Streptococcus pneumonia, Pseudomonas aeruginosa,	Relative frequency of amino acid triplets (RFAT), frequency difference of amino acid triplets (FDAT), amino acid composition (AC)	Hybrid model (CNN+LSTM)	Deng, L., Zhao, J., & Zhang, J. (2020, December). Predict the protein-protein interaction between virus and host through hybrid deep neural network. In *2020 IEEE International Conference on Bioinformatics and Biomedicine (BIBM)* (pp. 11-16). IEEE.	- Rich in terms of distribution of Amino acids
Hangyu, 2021	**Viruses:** SARS-CoV-2, SARS-CoV, HCoV-229E, HCoV-HKU1, HCoV-OC43, HCoVNL63, and MERS-CoV<br><br>**Hosts:** human, mouse, rat, dog, cat, camel, squirrel, cattle, chimpanzee, red junglefowl, rabbit, horse, monkey, rat, sheep, swine, and golden Syrian	Node2vec and Text2vec embeddings	MLP	Du, H., Chen, F., Liu, H., & Hong, P. (2021). Network-based virus-host interaction prediction with application to SARS-CoV-2. *Patterns*, *2*(5), 100242.	- Combining structural and content information together.

	hamster				
DeepVHPPI. 2021	**Viruses:** SARS-CoV-2, H1N1, Ebola<br><br>**Host:** Human	One hot encoding	Transformers + CNN	Lanchantin, J., Weingarten, T., Sekhon, A., Miller, C., & Qi, Y. (2021, August). Transfer learning for predicting virus-host protein interactions for novel virus sequences. In Proceedings of the 12th ACM Conference on Bioinformatics, Computational Biology, and Health Informatics (pp. 1-10).	-Simplified way to represent biological sequences<br><br>-High dimensional feature vectors may yield large feature vectors. Lack of relationship information about amino acids.
MTT, 2021	**Viruses:**<br>H1N1, Ebola, Arenaviridae, Parvoviridae, Floviviridae, Orthomyxoviridae, Pneumonoviridae, Paramyxoviridae, Retroviridae, Herpesviridae, Polyomaviridae, Adenoviridae, Flaviviridae, Poxviridae, Hepadnaviridae, Papilomaviridae<br><br>**Host:** Human	Random embeddings	LSTM	Dong, T. N., Brogden, G., Gerold, G., & Khosla, M. (2021). A multitask transfer learning framework for the prediction of virus-human protein–protein interactions. BMC bioinformatics, 22(1), 1-24.	
**Machine Learning Based Models**					
ML-AdVInfect, 2021	**Viruses:** Adenovirus<br><br>**Hosts:** 40 host species	kmer frequency (Denono), ,Relative frequency of amino acid triplets (RFAT), frequency difference of amino acid triplets (FDAT), Amino acid composition (AC), composition, transition and distribution of amino acid groups, Doc2vec (distributed-memory (DM)) , Autocovariance, Local descriptor (LD) based on composition (c), transition (T), and distribution (D)	SVM	Karabulut, O. C., Karpuzcu, B. A., Türk, E., Ibrahim, A. H., & Süzek, B. E. (2021). ML-AdVInfect: a machine-learning based adenoviral infection predictor. *Frontiers in Molecular Biosciences*, *8*.	- Rich in distribution of amino acid, contextual information etc.
Xiang Zhou et al., 2018	**Viruses:** 332 different types of viruses, Ebola virus,<br>Dengue virus, etc<br><br>**Hosts:** Homo sapiens , Mus musculus, Bos taurus, Rattus norvegicus, Non human Sus scrofa, Gallus gallus, Equus caballus, Drosophila melanogaster, Canis lupus familiaris, Arabidopsis thaliana, Escherichia coli, Streptococcus pneumonia, Pseudomonas aeruginosa, and Escherichia coli	Relative frequency of amino acid triplets (RFAT), frequency difference of amino acid triplets (FDAT), Amino acid composition (AC), composition, transition and distribution of amino acid groups	SVM	Zhou, X., Park, B., Choi, D., & Han, K. (2018). A generalized approach to predicting protein-protein interactions between virus and host. BMC genomics, 19(6), 69-77.	- Rich in distribution of amino acid
Denovo, 2022	**Viruses:**<br>Paramyxoviridae, Filoviridae, Bunyaviridae, Flaviviridae, Adenoviridae, Orthomyxoviridae, Chordopoxviridae,	kmer frequency based on the clusters obtained from amino acids properties like dipoles and volumes of side chains	SVM	Eid, F. E., ElHefnawi, M., & Heath, L. S. (2016). DeNovo: virus-host sequence-based protein–protein interaction prediction. *Bioinformatics*, *32*(8), 1144-1150.	- Lack of context information about amino acid

	Papillomaviridae, Herpesviridae, Retroviridae<br><br>**Host:** Human				
Xiaodi Yang, 2020	**Viruses:**<br>Influenza, Herpes viruses, Papillomaviruses, Saccharomyces cerevisiae, Human immunodeficiency virus, Yersinia, Zika virus, Hepatitis C virus, Bacillus, Dengue virus, Francisella tularensis, Measles virus<br><br>**Host:** Human	Doc2vec (distributed-memory (DM)) ,<br><br>for comparison: Autocovariance, Local descriptor (LD) based on composition (c), transition (T), and distribution (D)	**RF,** SVM, AB, MLP	Yang, X., Yang, S., Li, Q., Wuchty, S., & Zhang, Z. (2020). Prediction of human-virus protein-protein interactions through a sequence embedding-based machine learning method. *Computational and structural biotechnology journal*, *18*, 153-161.	
Barman, 2014	**Viruses:** Human immunodeficiency virus 1 (HIV-1), simian virus 40 (SV40), hepatitis B virus (HBV), hepatitis C virus (HCV), papilloma virus<br><br>**Host:** Human	Average domain-domain association score, Virus Methionine, virus seline and virus valine	RF, NB, SVM	Barman, R. K., Saha, S., & Das, S. (2014). Prediction of interactions between viral and host proteins using supervised machine learning methods. *PloS one*, *9*(11), e112034.	Advanced features [structure, gene ontology<br>(GO) or interaction domains] require prior knowledge that can be difficult and time-consuming to obtain
Saud Alguwaizani, 2018	**Viruses:** HCV, SARS virus, influenza A (H1N1) virus, human papillomavirus (HPV-16), and human immunodeciency virus HIV-1, other positive stranded RNA viruses<br><br>**Hosts:** Human, Mus musculus, Bos taurus, Rattus norvegicus, Sus scrofa, and Escherichia coli K-12.	Amino acid repeats, sum of squared length of single amino acid repeats (SARs), maximum of the sum of squared length of SARs in a window of 6 residues, composition of amino acids in 5 partitions of the protein sequence	SVM	Alguwaizani, S., Park, B., Zhou, X., Huang, D. S., & Han, K. (2018). Predicting interactions between virus and host proteins using repeat patterns and composition of amino acids. *Journal of healthcare engineering*, *2018*.	
