# Supplementary material for: MP-VHPPI: Meta predictor for viral host protein-protein interaction prediction in multiple hosts and viruses
Source: Front Med (Lausanne). 2022 Nov 16;9:1025887. doi: 10.3389/fmed.2022.1025887 (PMC9709337; doi:10.3389/fmed.2022.1025887)
Supplement: Supplementary file 2 [file Data_Sheet_2.PDF]

**AccNo**	**Hydrophobicity**	**Hydrophilicity**	**SideChainMass**
**A**	0.62	-0.5	15
**R**	-2.53	3	101
**N**	-0.78	0.2	58
**D**	-0.9	3	59
**C**	0.29	-1	47
**Q**	-0.85	0.2	72
**E**	-0.74	3	73
**G**	0.48	0	1
**H**	-0.4	-0.5	82
**I**	1.38	-1.8	57
**L**	1.06	-1.8	57
**K**	-1.5	3	73
**M**	0.64	-1.3	75
**F**	1.19	-2.5	91
**P**	0.12	0	42
**S**	-0.18	0.3	31
**T**	-0.05	-0.4	45
**W**	0.81	-3.4	130
**Y**	0.26	-2.3	107
**V**	1.08	-1.5	43

Table 1: Physiochemical values used in the APAAC encoder for hydrophobicity, hydrophilicity, and side chain mass.

Name	A	C	D	E	F	G	H	I	K	L	M	N	P	Q	R	S	T	V	W	Y
A	0	0.11	0.81	0.82	0.54	0.20	0.69	0.40	0.89	0.40	0.37	0.318	0.19	0.37	1	0.09	0.22	0.27	0.73	0.55
C	0.11	0	0.84	0.83	0.43	0.32	0.66	0.30	0.88	0.30	0.27	0.324	0.15	0.34	1	0.17	0.23	0.16	0.63	0.45
D	0.72	0.74	0	0.12	0.92	0.69	0.43	0.84	0.24	0.84	0.81	0.56	0.65	0.58	0.29	0.66	0.64	0.79	1	0.83
E	0.79	0.78	0.13	0	0.93	0.77	0.40	0.86	0.14	0.85	0.83	0.599	0.68	0.59	0.23	0.72	0.68	0.82	1	0.83
F	0.50	0.40	0.97	0.91	0	0.69	0.66	0.12	0.90	0.13	0.16	0.541	0.42	0.45	1	0.54	0.49	0.25	0.20	0.17
G	0.20	0.31	0.77	0.80	0.72	0	0.76	0.59	0.89	0.59	0.55	0.381	0.32	0.46	1	0.15	0.27	0.46	0.92	0.72
H	0.89	0.83	0.62	0.54	0.90	1	0	0.84	0.56	0.84	0.82	0.754	0.77	0.71	0.69	0.86	0.83	0.83	0.98	0.82
I	0.40	0.29	0.94	0.89	0.13	0.59	0.65	0	0.89	0.01	0.05	0.457	0.31	0.38	1	0.44	0.39	0.13	0.33	0.21
K	0.88	0.87	0.27	0.14	0.95	0.9	0.43	0.89	0	0.89	0.87	0.667	0.75	0.63	0.15	0.82	0.75	0.88	1	0.84
L	0.40	0.29	0.94	0.89	0.13	0.59	0.65	0.01	0.89	0	0.06	0.452	0.30	0.37	1	0.44	0.39	0.13	0.34	0.20
M	0.38	0.27	0.93	0.87	0.18	0.56	0.64	0.05	0.88	0.06	0	0.447	0.28	0.37	1	0.41	0.35	0.12	0.39	0.25
N	0.42	0.42	0.83	0.83	0.76	0.51	0.78	0.61	0.89	0.60	0.58	0	0.26	0.17	1	0.36	0.36	0.50	0.94	0.64
P	0.22	0.17	0.85	0.83	0.51	0.37	0.69	0.36	0.87	0.35	0.32	0.231	0	0.22	1	0.19	0.16	0.24	0.72	0.48
Q	0.51	0.46	0.90	0.86	0.67	0.64	0.76	0.53	0.88	0.51	0.50	0.181	0.27	0	1	0.46	0.38	0.46	0.83	0.52
R	0.91	0.90	0.30	0.22	0.97	0.92	0.49	0.92	0.14	0.92	0.90	0.69	0.79	0.66	0	0.86	0.80	0.91	1	0.85
S	0.1	0.18	0.80	0.81	0.62	0.17	0.71	0.47	0.88	0.47	0.44	0.289	0.18	0.35	1	0	0.17	0.34	0.82	0.61
T	0.25	0.26	0.83	0.81	0.60	0.31	0.73	0.45	0.86	0.45	0.40	0.315	0.15	0.32	1	0.18	0	0.34	0.81	0.59
V	0.27	0.16	0.9	0.86	0.26	0.47	0.64	0.13	0.88	0.13	0.12	0.38	0.21	0.33	1	0.32	0.30	0	0.47	0.31
W	0.65	0.56	1	0.93	0.19	0.82	0.67	0.30	0.89	0.30	0.34	0.631	0.55	0.53	0.96	0.68	0.63	0.41	0	0.20
Y	0.58	0.47	1	0.93	0.20	0.78	0.67	0.23	0.90	0.21	0.26	0.512	0.44	0.40	0.99	0.61	0.55	0.32	0.24	0

Table 2: Distance or Content matrix computed by Schneider and Wrebe on the basis of 4 different physiochemical properties i.e., hydrophobicity, hydrophilicity, polarity, chain mass.

Name	A	R	N	D	C	Q	E	G	H	I	L	K	M	F	P	S	T	W	Y	V
A	0	112	111	126	195	91	107	60	86	94	96	106	84	113	27	99	58	148	112	64
R	112	0	86	96	180	43	54	125	29	97	102	26	91	97	103	110	71	101	77	96
N	111	86	0	23	139	46	42	80	68	149	153	94	142	158	91	46	65	174	143	133
D	126	96	23	0	154	61	45	94	81	168	172	101	160	177	108	65	85	181	160	152
C	195	180	139	154	0	154	170	159	174	198	198	202	196	205	169	112	149	215	194	192
Q	91	43	46	61	154	0	29	87	24	109	113	53	101	116	76	68	42	130	99	96
E	107	54	42	45	170	29	0	98	40	134	138	56	126	140	93	80	65	152	122	121
G	60	125	80	94	159	87	98	0	98	135	138	127	127	153	42	56	59	184	147	109
H	86	29	68	81	174	24	40	98	0	94	99	32	87	100	77	89	47	115	83	84
I	94	97	149	168	198	109	134	135	94	0	5	102	10	21	95	142	89	61	33	29
L	96	102	153	172	198	113	138	138	99	5	0	107	15	22	98	145	92	61	36	32
K	106	26	94	101	202	53	56	127	32	102	107	0	95	102	103	121	78	110	85	97
M	84	91	142	160	196	101	126	127	87	10	15	95	0	28	87	135	81	67	36	21
F	113	97	158	177	205	116	140	153	100	21	22	102	28	0	114	155	103	40	22	50
P	27	103	91	108	169	76	93	42	77	95	98	103	87	114	0	74	38	147	110	68
S	99	110	46	65	112	68	80	56	89	142	145	121	135	155	74	0	58	177	144	124
T	58	71	65	85	149	42	65	59	47	89	92	78	81	103	38	58	0	128	92	69
W	148	101	174	181	215	130	152	184	115	61	61	110	67	40	147	177	128	0	37	88
Y	112	77	143	160	194	99	122	147	83	33	36	85	36	22	110	144	92	37	0	55
V	64	96	133	152	192	96	121	109	84	29	32	97	21	50	68	124	69	88	55	0

Table 3: Distance or Content matrix computed by Grantham on the basis of 4 different physiochemical properties i.e., hydrophobicity, hydrophilicity, polarity, chain mass.
